# An intragenic mutagenesis strategy in *Physcomitrella patens* to preserve intron splicing

**DOI:** 10.1038/s41598-017-05309-w

**Published:** 2017-07-11

**Authors:** Ako Eugene Ako, Pierre-François Perroud, Joseph Innocent, Viktor Demko, Odd-Arne Olsen, Wenche Johansen

**Affiliations:** 1 Inland Norway University of Applied Sciences, Holsetgata 31, N-2318 Hamar, Norway; 2 Philipps University Marburg, Plant Cell Biology II, Karl-von-Frisch-Str. 8, 35043 Marburg, Germany; 3 Norwegian University of Life Sciences, P.O. Box 5003, N-1432 As, Norway

## Abstract

Gene targeting is a powerful reverse genetics technique for site-specific genome modification. Intrinsic homologous recombination in the moss *Physcomitrella patens* permits highly effective gene targeting, a characteristic that makes this organism a valuable model for functional genetics. Functional characterization of domains located within a multi-domain protein depends on the ability to generate mutants harboring genetic modifications at internal gene positions while maintaining the reading-frames of the flanking exons. In this study, we designed and evaluated different gene targeting constructs for targeted gene manipulation of sequences corresponding to internal domains of the DEFECTIVE KERNEL1 protein in *Physcomitrella patens*. Our results show that gene targeting-associated mutagenesis of introns can have adverse effects on splicing, corrupting the normal reading frame of the transcript. We show that successful genetic modification of internal sequences of multi-exon genes depends on gene-targeting strategies which insert the selection marker cassette into the 5′ end of the intron and preserve the nucleotide sequence of the targeted intron.

## Introduction

Engineering loss- and gain-of-function alleles by reverse genetic studies is a powerful approach for dissecting the biochemical function of a gene product *in vivo*. Several different tools and resources are available for reverse genetic approaches in plants, including RNA-mediated interference (RNAi)^1^, virus-induced gene silencing (VIGS)^2^, targeted induced local lesions in genomes (TILLING)^3^, clustered regularly interspaced short palindromic repeats (CRISPR)/CRISPR-associated protein 9 (Cas9) (CRISPR/Cas9)^4^ and gene targeting (GT)^5^. Gene targeting is a technique for introducing site-specific genome modifications at an endogenous locus via homologous recombination (HR) with exogenous DNA. Because of its site-specific precision and the almost unlimited types of DNA modifications that can be introduced, e.g. deletions, inversions, insertions and point mutations, GT remains one of the most powerful methods available to analyze gene function. However, in angiosperm plants as well as in most higher eukaryotes, GT is inefficient due to the fact that the frequency of random DNA integration by illegitimate recombination (IR) exceeds that of targeted integration by HR by several orders of magnitude^6, 7^. Mosses, particularly *Physcomitrella patens*, where GT is as efficient as in *Saccharomyces cerevisiae* ^5, 8^, and much more efficient than in mouse ES cells^9^ and in the green alga *Chlamydomonas reinhardtii* ^10^, represent an exception to this rule in plants. Recently, it was demonstrated that GT-efficiency could be significantly improved in moss using the CRISPR/Cas9 system^11, 12^. Highly efficient GT in *P*. *patens* also permits complex *in vivo* assembly of multiple DNA fragments with overlapping sequences of as short as 12 bp^13^. This also expands the potential of this model organism for biotechnological applications similar to that of yeast^14, 15^.

The intrinsic efficiency of HR in *P*. *patens* has made GT the predominant method for functional genomics in this species. Knock-out of a gene in *P*. *patens* is routinely achieved using a construct harboring a positive selection marker gene, such as those conferring neomycin (*neo*) or hygromycin (*hpt*) resistance, flanked by targeting sequences (TS) homologous to the 5′ and 3′ flanking regions of the sequence to be modified^16^. Upon transformation, homologous exchange between the TSs in the vector and their genome counterparts results in a targeted gene deletion and insertion of the marker cassette. Constructs for homology-dependent gene insertion (knock-in) are similar in design to knock-out vectors, except that the sequence to be introduced is fused in-frame with one of the TSs in the vector. The selection marker gene used in GT vectors can be flanked by *loxP* sequences that later can be exploited to obtain marker-free mutant lines by Cre/lox-mediated recombination^17–22^. The site-specific Cre recombinase, which is introduced by transformation of a Cre-expressing vector^23^, specifically recognizes the recombining *loxP* sites and mediates deletion of the marker gene, leaving a singular *loxP* site at the recombined locus^23^.

The majority of HR-promoted gene disruptions in *P*. *patens* represent single gene knock-out mutants generated to study gene-function relationships, in addition to gene tagging experiments allowing *in vivo* monitoring of target gene expression and fusion protein localization. However, since most eukaryotic proteins are composed of multiple domains, often with specific functions, mutants harboring specific partial gene deletions are often desirable for functional studies. Design of the vector for targeted gene manipulations of individual domains is a critical step, and requires knowledge of the intron-exon borders in order to precisely modify the nucleotide sequence corresponding to the domain of interest while preserving the reading frame of the gene. In addition, deletion of individual domains located internally in a protein and/or for which the coding sequence spans two or more exons are further complicated by the insertion of the selection marker into intronic sequences, which potentially can interfere with splicing. *P*. *patens* mutants harboring partial gene deletions have been reported, the deleted domains being either located at the N- or C-terminus of the protein^24^. Although common practice in yeast^25, 26^ and mice^27, 28^, reports of targeted partial deletion of sequences corresponding to internal protein domains are rare in plants.

**Figure 1:**
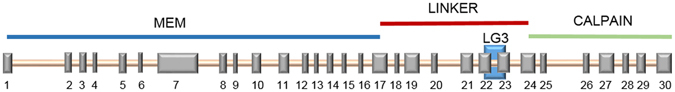
Intron-exon structure of the *Physcomitrella patens DEK1* gene. *DEK1* ORF consists of 30 exons (grey rectangles) encoding the MEM domain (blue line), the LINKER segment (red line), the LG3 domain (blue rectangle) and the CALPAIN domain (green line). The numbers identify the exons of the *DEK1* ORF.

DEK1 is a large multi-domain membrane-anchored cysteine protease of approximately 240 kDa composed of an N-terminal membrane domain (MEM) harboring 23 transmembrane segments linked to the C-terminal calpain domain (CysPc-C2L) by a Linker segment, containing a Laminin-G3 (LG3) domain (Fig. 1)^29^. Previously, we created the *P*. *patens DEK1* knockout mutant (*Δdek1*), in which the entire 14,569 bp *DEK1* gene of 30 coding exons was deleted using GT. The *Δdek1* mutant develops protonemata, filamentous cells exhibiting polar tip growth; however, leafy gametophores fail to form due to an early arrest of bud development^30^. The same phenotype is obtained in a mutant (*dek1* ^*0*^)^29^ in which the active site cysteine residue of the DEK1 protease core domain is substituted with serine, a mutation that also inactivates DEK1 calpain activity *in vitro* ^31^. Thus, mutant plants with calpain-null alleles display a clearly detectable phenotype with filamentous growth without gametophores. A much milder phenotype is observed in the *P*. *patens dek1Δlg3* mutant, generated by in-frame removal of the sequence corresponding to the DEK1-LG3 domain. This mutant forms reduced gametophores with narrow phyllids lacking several features of the WT phyllids, including the midrib^29^.

In this report, we evaluate the performance of different *DEK1* GT genome editing constructs for the production of mutants harboring specific internal gene deletions and insertions in *P*. *patens*. Generation of mutagenized *DEK1* transcripts with an intact open-reading frame was used as a criterion for successful gene modification. Our results show that intragenic mutagenesis in *P*. *patens* is critically dependent on a vector design that conserves intron splicing signals. We propose useful guidelines and discuss important considerations in the design of GT vectors for targeting internal sequences of multi-exon genes in *P*. *patens*.

## Results

### Design of gene targeting vectors for internal exon deletions

**Figure 2:**
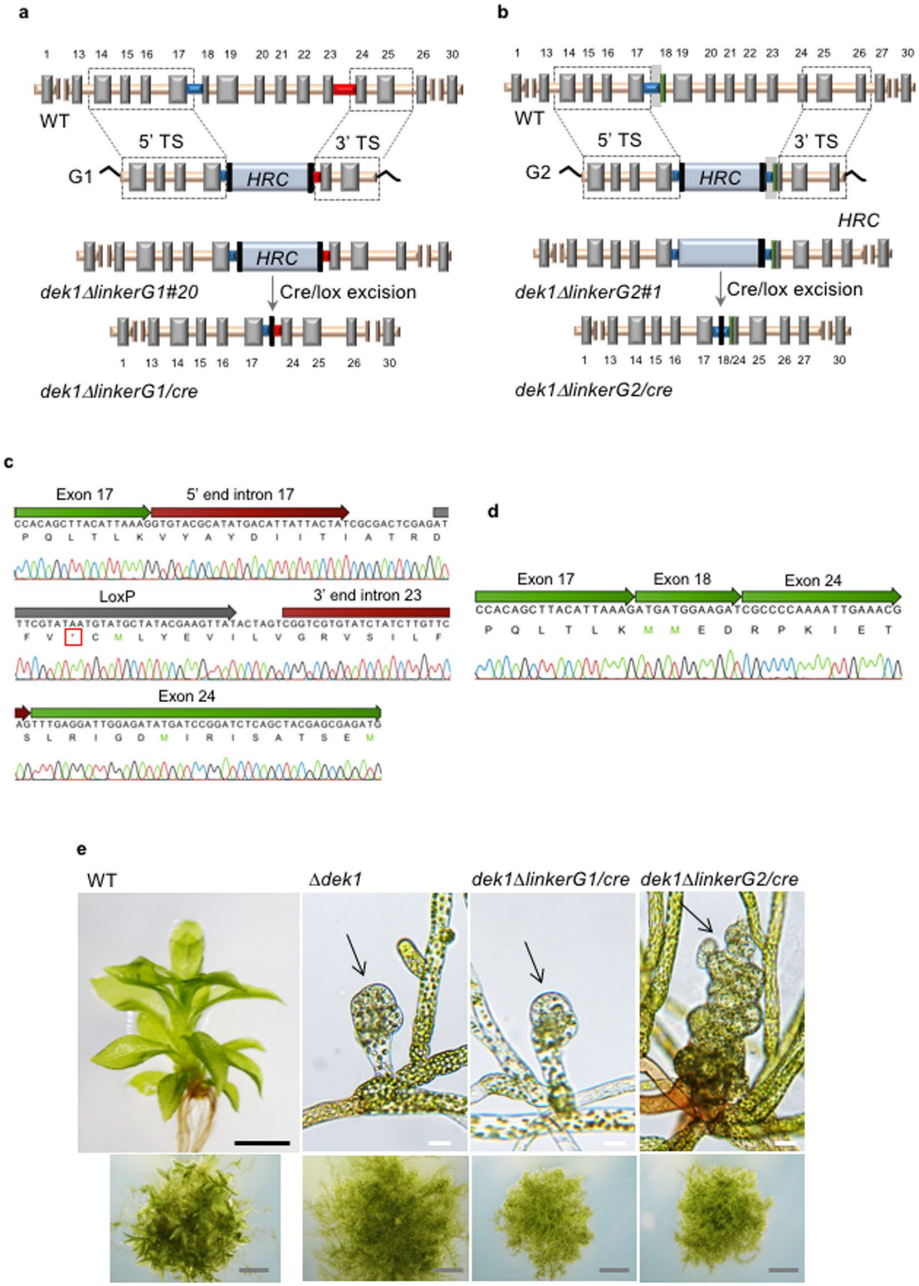
*DEK1-Linker* knock-out. Schematic representation of the *DEK1-Linker* knock-out strategies using the (**a**) G1 and (**b**) G2 vectors and elimination of the hygromycin resistance cassette (*HRC*) (light blue box) by Cre/loxP-mediated recombination. The grey boxes correspond to exons in the *P*. *patens DEK1* gene. Introns are colored in orange, with the exception of introns 17 (blue) and 23 (red). The 5′ and 3′ targeting sequences (TS) are boxed. The *loxP* sites are shown as vertical black lines. Numbers identify the exons of the *DEK1* gene. (**c**) cDNA sequencing electropherogram of the *dek1∆linkerG1*/*cre DEK1* transcript showing deletion of exon 18–23 and retention of the *loxP*-harboring hybrid intron 17/23, creating a premature termination code (red box). (**d**) cDNA sequencing electropherogram of the *dek1∆linkerG2*/*cre DEK1* transcript showing in-frame fusion of exons 17, 18 and 24. (**e**) Phenotype of 3-week-old *dek1∆linkerG1*/*cre* and *dek1∆linkerG2*/*cre* plants. For comparison, wild-type (WT) and *∆dek1* are also shown. WT plants with developing gametophores. *dek1∆linkerG1*/*cre* and *dek1∆linkerG2*/*cre* mutant plants are showing filamentous growth and aborted buds (arrows). *dek1∆linkerG1*/*cre* bud development stops at an early stage leading to small arrested buds, similar to *∆dek1*; the buds of *dek1∆linkerG2*/*cre* mutant continue to divide, giving rise to larger disorganized bud structures. Scale bars: grey = 2 mm, black = 500 μm and white = 25 μm.

Two different GT vector designs G1 and G2 were tested to delete the *Linker* encoded by *DEK1* exons 17–24 (Fig. 1) in order to create a *P*. *patens* (*Pp*) mutant expressing a truncated DEK1 protein lacking the internal Linker segment. The G1 vector was designed to insert the 5′ and 3′ ends of the *loxP*-flanked *Hygromycin Resistance Cassette* (*HRC*) into *DEK1* introns 17 and 23, respectively. With this vector, we expected to produce a mutant with targeted deletion of a 3198 bp *DEK1* fragment (nucleotides (nt) 8347–11544; XP_001774206.1) of the *Linker* sequence. In addition, transformation was expected to yield a *DEK1* hybrid intron composed of the *HRC* flanked by the 5′ and 3′ ends of introns 17 and 23, respectively (Fig. 2a). The G2 vector was designed to insert the *loxP*-flanked *HRC* into *DEK1* intron 17 without affecting the intron nucleotide composition (Fig. 2b). To this end, the G2 5′ targeting sequence (TS) contained ~1.3 kb genomic sequence (nt 7249–8404) ending in *DEK1* intron 17. The ~1.2 kb 3′ TS was a mutagenized genomic sequence (nt 8402–12893, Δ8462–11718) composed of *DEK1* nucleotides from intron 17 followed by the first 12 nucleotides of exon 18 fused in frame with the 3′ end of exon 24, to intron 26. Using the G2 vector for transformation, we expected targeted deletion of the *DEK1* sequence from within exon 18 to within exon 24, in addition to insertion of the *HRC* into the full-length intron 17 (Fig. 2b).

Following transformation of wild-type *P*. *patens* protoplasts with the G1 and G2 vectors, six *dek1ΔlinkerG1* and one *dek1ΔlinkerG2* hygromycin-resistant (Hyg^R^) on-locus targeted mutant lines, respectively, were identified by PCR (data not shown). These plants all displayed a phenotype similar to the *dek1* ^*0*^ mutant^29^ with filamentous growth and lacking gametophores, as shown for the *dek1ΔlinkerG1#20* and *dek1ΔlinkerG2#1* lines in Supplementary Fig. S1. Insertion of the 1.8 kb *HRC* in an intron may potentially cause a null mutant phenotype by interfering with posttranscriptional modification of the transcript, preventing expression of the active protein. This was subsequently confirmed by reverse-transcription (RT)-PCR analysis using primers annealing outside TS and within the resistance marker cassette, with positive amplification signals for the presence of the *HRC* in the cDNA amplified from the two mutant lines (data not shown). Thus, in order to eliminate the resistant marker from the *DEK1* locus, *dek1ΔlinkerG1#20* and *dek1ΔlinkerG2#1* were subjected to Cre/loxP-mediated recombination, resulting in the hygromycin-sensitive (Hyg^S^) lines named *dek1ΔlinkerG1*/*cre* and *dek1ΔlinkerG2*/*cre*. The mutants were verified by Southern hybridization confirming targeted deletion of the *Linker*, no sign of secondary non-specific integration, and loss of the *HRC* from the locus (Supplementary Fig. S2a). To investigate *DEK1* transcript processing in *dek1ΔlinkerG1*/*cre* and *dek1ΔlinkerG2*/*cre*, a cDNA spanning exon 11 to exon 27 was amplified by RT-PCR using primers annealing outside the TSs, resulting in the generation of a single PCR product (Supplementary Fig. S2b) which was subsequently sequenced (Fig. 2c,d). Analysis of the *dek1ΔlinkerG1*/*cre* cDNA revealed retention of the hybrid intron 17/23 in the truncated *DEK1* transcript (Fig. 2c). Retention of this intron introduced a premature stop codon 5′ to the calpain coding sequence (Fig. 2c), caused by a frame-shift mutation. In contrast, analysis of the *dek1ΔlinkerG2*/*cre* cDNA confirmed the expected splicing product of the truncated *DEK1* transcript, with the 5′ end of exon 18 fused in frame with the 3′ end of exon 24 (Fig. 2d). Consistent with the molecular data, *dek1ΔlinkerG1*/*cre* displayed a typical DEK1 calpain-null mutant phenotype^29, 30^ with lack of gametophores and aborted buds (Fig. 2e). In contrast, *dek1ΔlinkerG2*/*cre* mutant buds divide beyond those of *Δdek1*, forming larger disorganized buds with abortive growth (Fig. 2e), suggesting the presence of residual levels of DEK1 calpain activity in the mutant^29^.

### Design of gene targeting vectors for internal exon insertions

**Figure 3:**
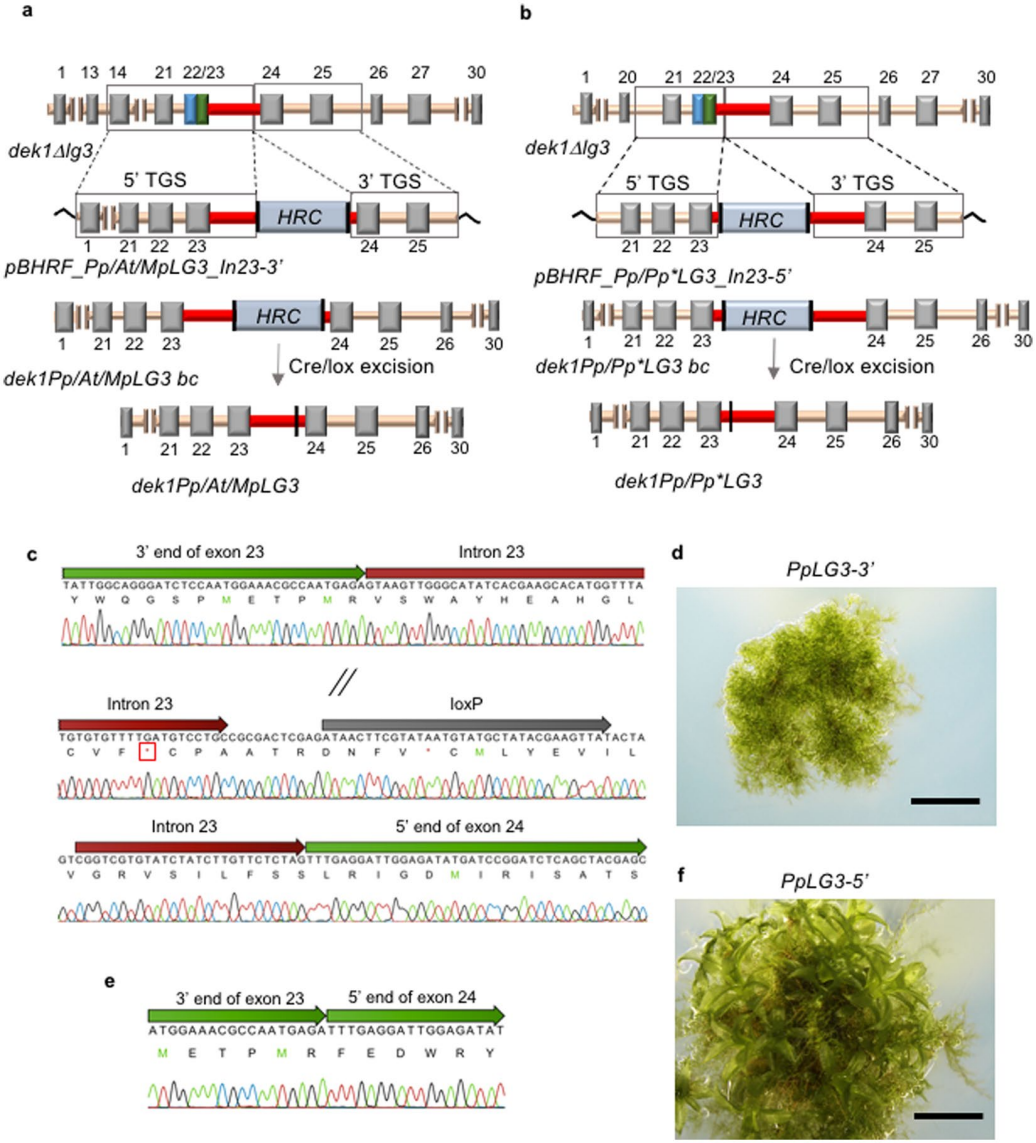
*DEK1-LG3* knock-in. Schematic representation of the *DEK1-LG3* knock-in strategies using the (**a**) In23-3′ and (**b**) In23-5′ vectors, and elimination of the *hygromycin* resistance cassette (*HRC*) (light blue box) by Cre/loxP-mediated recombination. The grey, blue and green boxes correspond to the exons in the *P*. *patens DEK1* gene. The blue and green boxes represent the fused exon 22 and 23, respectively, of the *dek1Δlg3* mutant. Introns are colored in orange, with the exception of intron 23, which is colored red. The 5′ and 3′ targeting sequences (TS) are boxed. The *loxP* sites are shown as vertical black lines. The numbers correspond to the exons of the *DEK1* gene. For simplicity, the *PpLG3-M1*, *PpLG3-M2* and *PpLG3-M3* mutant lines are collectively denoted as Pp*LG3. (**c**) cDNA sequencing electropherogram of the *PpLG3-3*′ *DEK1* transcript showing retention of the *loxP*-harboring intron 23, resulting in a premature stop codon (red box). The slanted lines denote omitted sequences due to space limitation. (**d**) Phenotype of 3-week-old *PpLG3-3*′ mutant plant showing filamentous growth without gametophores. (**e**) cDNA sequencing electropherogram of the *PpLG3-5*′ *DEK1* transcript showing in-frame fusion of exon 23 and exon 24. (**f**) Phenotype of 3-week-old *PpLG3-5*′ mutant plant showing well-developed gametophores, demonstrating successful complementation of the *dek1Δlg3* mutant phenotype. Scale bars: 2 mm.

We next tested two different GT vector designs (In23-5′ and In23-3′) for knock-in of wild-type, heterologous and mutated *DEK1-LG3* sequences into the *Pp dek1Δlg3* mutant. The LG3 domain is encoded within exon 22 and 23 of the *PpDEK1* gene (Fig. 1), and the *dek1Δlg3* mutant contains a deletion in the genomic region spanning the LG3 domain^29^. Both for the In23-5′ and the In23-3′ designs, the complementing *LG3* sequence is fused in frame with the 5′ TS, and the *loxP* flanked *HRC* are targeted to intron 23, however the position of the *HRC* insertion within intron 23 differed between the two (Fig. 3a,b).

In the In23-3′ design, the *HRC* was targeted to the end of intron 23, 27 bp upstream of the 3′ end of the intron (Fig. 3a and Supplementary Fig. S3a). In this set of experiments, wild-type genomic *LG3* sequences from *Pp*, *Arabidopsis thaliana* (*At*) and *Marchantia polymorpha* (*Mp*) were targeted to the *Pp DEK1ΔLG3* locus. In the In23-5′ design, the *HRC* was targeted to the start of intron 23, 28 bp downstream of the 5′ end of the intron (Fig. 3b and Supplementary Fig. S3b), and in this set of experiments, we targeted the wild-type and three mutated *Pp LG3* versions (LG3/M1, LG3/M2 and LG3/M3, see Methods) to the *DEK1ΔLG3* locus. Following transformation of *dek1Δlg3* protoplasts with the vectors built using the In23-3′ design, we obtained 40 *PpLG3-3*′, 105 *MpLG3* and 74 *AtLG3* Hyg^R^ lines in which four, five and one, respectively were on-locus targeted as demonstrated by PCR genotyping (data not shown). The *HRC* was subsequently removed from one representative of each of the *PpLG3-3*′, *AtLG3* and *MpLG3* lines using Cre/loxP-mediated recombination. *DEK1* cDNA of marker-free *PpLG3-3*′/*cre*, *AtLG3*/*cre* and *MpLG3*/*cre* lines were amplified and analyzed as described in the preceding section, confirming insertion of the wild-type (Supplementary Fig. S3c(i)) and heterologous *LG3* sequences (Supplementary Fig. S3c(ii,iii)) at the *DEK1* locus. In all lines, however, intron 23 was retained in the *DEK1* transcript, as shown by DNA sequencing of the *DEK1* cDNA amplification-product of line *PpLG3-3*′/*cre* in Fig. 3c. Retention of the *loxP*-harboring intron in the *DEK1* transcript caused a frame-shift mutation leading to a premature termination codon and a putative truncated DEK1 protein lacking the catalytic calpain domain. Consistently, all mutant lines displayed a calpain-null mutant phenotype similar to *Δdek1* and *dek1* ^*0*^ with the lack of gametophores as shown for *PpLG3-3*′/*cre* in Fig. 3d, and for *AtLG3*/*cre* and *MpLG3*/*cre* in Supplementary Fig. S3d. In addition to retention of *Pp DEK1* intron 23, the *AtLG3*/*cre DEK1* transcript displayed additional pre-mRNA splicing errors, specifically the loss of 64 exonic nucleotides corresponding to 61 nucleotides at the 3′ end of *At DEK1* exon 21 followed by the first three nucleotides of *At* exon 22 (Supplementary Fig. S3c(ii)). In contrast, sequencing of the *DEK1* cDNA from *MpLG3*/*cre* confirmed proper splicing of the heterologous *Mp LG3* sequence (Supplementary Fig. S3c(iii)).

Transformation of *dek1Δlg3* protoplasts with vectors built using the In23-5′ strategy (Fig. 3b and Supplementary Fig. S3b), yielded 3 *PpLG3-5*′, 3 *PpLG3-M1*, 5 *PpLG3-M2* and 8 *PpLG3-M3* Hyg^R^ on-locus targeted lines (data not shown) with the *HRC* from one representative line for each having been removed by Cre/lox-mediated excision. Sequence analysis of *DEK1* cDNA amplified from marker-free lines showed in-frame re-targeting of the *Pp LG3* sequences and proper splicing at the locus, as shown for mutant *PpLG3-5*′/*cre* in Fig. 3e. Sequencing also confirmed the presence of the desired *LG3* mutations in the *DEK1* transcript of *PpLG3-M1*/*cre*, *PpLG3-M2*/*cre* and *PpLG3-M3*/*cre* (Supplementary Fig. S3e). The *PpLG3-5*′/*cre* (Fig. 3f) and *PpLG3-M1*/*cre*, *PpLG3-M2*/*cre* and *PpLG3-M3*/*cre* mutants (Supplementary Fig. S3f) all developed gametophores with fully expanded phyllids similar to wild-type plants, and clearly distinguishable from the narrow phyllid phenotype of the *dek1Δlg3* mutant^29^ (Supplementary Fig. S3f), thus confirming successful complementation at the *DEK1ΔLG3* locus using the In23-5′ vector design.

## Discussion

*In vivo* genetic engineering of internal exonic sequences is complicated by the targeting of intronic sequences with a selection marker cassette, which may interfere with post-transcriptional modifications, disrupting gene expression. Although the resistance marker cassette can be efficiently eliminated from the targeted intron using e.g. the Cre/lox system, one *loxP* site will be retained at the locus following recombination. In this study, we show that successful genetic modifications of internal protein domains is dependent on the critical and rational design of the GT construct in order to avoid gene targeting-associated mutagenesis of introns having detrimental effects on splicing of the targeted transcript.

Two different knock-out vectors (G1 and G2) for targeted deletion of the internal *DEK1 Linker* were constructed and tested in *P*. *patens* plants. Transformation with both vectors resulted in mutants with targeted deletion of the *Linker*. However, while the G2 strategy resulted in a mutant plant (*dek1ΔlinkerG2*/*cre*) expressing a *DEK1* transcript with in-frame deletion of the *Linker*, the G1 strategy resulted in a mutant plant (*dek1ΔlinkerG1*/*cre*) expressing a *DEK1* transcript containing a frame-shift mutation leading to a premature termination code. The frame-shift mutation was caused by retention of the *loxP*-harboring hybrid intron 17/23, impairing *DEK1* pre-mRNA splicing. Thus, the G1, but not the G2 strategy resulted in the introduction of mutations causing splicing defects of the targeted intron. Three structural elements are used to correctly excise introns from primary transcripts. These include the 5′ and 3′ splicing sites spanning the 5′ exon/intron and the 3′ intron/exon borders respectively, and the internal branch point (BP) sequence located 19–50 nt upstream of the 3′ splicing site^32^. The hybrid *DEK1* 17/23 intron of *dek1ΔlinkerG1*/*cre* contains both the heterologous 5′ and 3′ splicing sites, but lacks the internal sequences of the native introns since only the 5′ and 3′ portions of intron 17 and 23, respectively, are retained after recombination between the G1 donor vector and the *DEK1* locus (Fig. 2a). This likely resulted in a loss of internal splicing signals, e.g the BP sequence within the targeted intron, thus abolishing splicing of exon 17 and 24. In contrast, splicing of the mutagenized, *loxP*-harboring *DEK1* intron 17 of the *dek1ΔlinkerG2*/*cre* mutant occurred properly. In this intron, no nucleotides were deleted during targeted insertion of the *HRC*. These results demonstrate that when designing GT knock-out vectors aimed at deleting internal exonic sequences, a strategy that preserves the native sequence of the targeted intron(s) and which minimizes or eliminates the loss of intronic nucleotides, should be chosen. This is achieved by using a strategy (G2) as described in this study; the selection marker cassette in the donor vector is placed within the sequence of the native intron (Fig. 2b). The 3′ end of this intron is followed by a sequence of the adjacent (3′) exon, which is fused in frame to the 3′ TS starting with a downstream exon. This design thus creates a heterologous exon at the targeted locus, containing a mutagenized sequence harboring a deletion of the genomic sequence intervening the original exons of the heterologous exon. Using this strategy, intronic sequences are preserved.

We also designed and evaluated two different GT knock-in vectors (termed In23-5′ and In23-3′) for targeted insertion of wild-type, heterologous and mutated versions of *DEK1-LG3* sequences into the *P*. *patens dek1Δlg3* mutant. Knock-in of a gene fragment is commonly performed in genetic complementation tests to confirm that a mutant phenotype is caused by a specific gene deletion, or to introduce modified versions of the gene for functional studies. In complementation tests where the outcome of the gene modification cannot be predicted, possibly giving a phenotype indistinguishable from that of the background line used for transformation, a positive selection marker is needed to identify transformed plants. Similar to gene knock-out approaches, knock-in of DNA fragments into internal regions of a gene is complicated by the necessity of targeting the selection marker gene to introns. Transformation of *dek1Δlg3* protoplasts with In23-5′-based vectors, where the resistant marker was inserted 28 bp downstream of the start of intron 23, expressed *DEK1* transcripts in which the reading-frame was maintained, and the plants reverted to a wild-type gametophore phenotype, confirming successful knock-in of *DEK1-LG3* into the *DEK1ΔLG3* locus. In contrast, *dek1Δlg3* protoplasts transformed with In23-3′-based vectors, where the resistance marker was inserted 27 bp upstream of the end of intron 23, expressed *DEK1* transcripts containing the *loxP-*harboring intron 23. These plants displayed a calpain-null mutant phenotype, as expected for DEK1 plants lacking the catalytic calpain domain. These results demonstrate that *HRC* insertion into the 3′ end, but not the 5′ end, of *DEK1* intron 23 impairs splicing, suggesting that a residing *loxP* sequence located 3′, but not 5′, corrupts the internal splicing signals of the intron. To explore this possibility further, we used the ERISdb program (http://lemur.amu.edu.pl/share/ERISdb/home.html)^33^, which predicts splice sites and splicing signals in various plant species, including *P*. *patens*. Using this tool, we predicted a putative branch site 36 bp upstream of the 3′ splice site of *DEK1* intron 23, close to the position in which the *loxP* sequence is retained in the mis-spliced mutagenized *DEK1* intron 23. The BP sequence is important for splicing in plants^34^, the sequence between the BP and the 3′ splicing site, tending to be U-rich, strongly influences splicing efficiency and splice site selection in plants^35^. Thus, insertion of a *loxP* site into this region may have corrupted the splicing signal of the intron, causing intron 23 to evade spliceosome activity. Our results demonstrate that selection of the marker cassette insertion point within the intron is important when designing GT vectors. BP sequences are normally located 19–50 nucleotides upstream of the 3′ splicing site. In order to avoid interfering with essential BP signals, we recommend to insert the selection marker close to the 5′ end of the intron and, if possible, at least 50 nucleotides upstream of the 3′ end of the intron. Correspondingly, the G2 donor vector that was successfully used to create the mutant plant expressing the *DEK1* transcript harboring an in-frame deletion of the *Linker*, was also designed to insert the resistant marker to the 5′ end of the targeted intron. Thus, our results show that targeting of the resistance marker cassette to the 5′ end of the intron should be preferred. It should be mentioned that choosing an appropriate position to insert the marker cassette may be problematic if the targeted intron is small. In such case, identifying a position where GT-associated mutagenesis (*loxP* retention) will not affect splicing, may be challenging. Therefore, if the insertion position within the gene is not critical for the experiment, we recommend the targeting of larger sized introns.

Recently it was shown that the *piggyBAC* transposon could be used for the precise and effective excision of selection marker cassettes from the genome without leaving any residual ectopic sequences both in mammalian cells^36^ and rice^37, 38^. The transposon, which derives from the cabbage looper *Trichoplusia ni* ^39^, integrates into the host genome at TTAA elements and has been used efficiently for transgenesis in animals^40^. The *piggyBAC* transposon thus offers an alternative method to the Cre/loxP system for elimination of a selection marker cassette from genomes. However, with the exception that the piggyBAC system has yet to be tested in *P*. *patens*, the main limitation of the system is the requirement for specific insertion into a TTAA sequence, maxing the system less flexible especially for precise site-directed mutagenesis.

An attractive and powerful alternative to classical GT methods in *P*. *patens* is the CRISPR/Cas9 system^12^. In *P*. *patens* both transformation frequency and GT efficiency have been found to be significantly enhanced using CRISPR/Cas9 when compared to the classical method using GT donor vectors only^11^. However, no reports have been published on the use of the CRISPR/Cas9 system for precise partial deletions or insertions of gene sequences at internal positions. Ultimately, the CRISPR/Cas9 approach may be efficient enough to bypass the need of the resistance cassette for such partial deletions. But for now, as with classical GT approaches, insertion of a resistance marker cassette to internal intronic sequences is necessary in order to select for transformants receiving the donor DNA. Thus, the results reported in the present study would also apply to the design of the donor vector using the CRISPR/Cas9 system.

In this study, we also demonstrate that *P*. *patens* has the capacity to properly splice a genomic fragment from the liverwort *M*. *polymorpha*, suggesting conservation of splicing signals between mosses and liverworts. In contrast, the genomic *A*. *thaliana DEK1* sequence in *P*. *patens* is incorrectly processed, suggesting the possibility that the moss spliceosome machinery does not properly recognize the splicing signals from dicot plants. This may stem from differences in the UA content of the introns; *P*. *patens* introns display a significantly lower UA content, 60%, compared to 67% in *A*. *thaliana* ^41^. Notably, Perroud and Quatrano^42^ showed that *P*. *patens* correctly splices the *BRK1* gene from *A*. *thaliana*, indicating that accurate processing of certain *A*. *thaliana* introns occurs in the moss.

It is worth noting that another knock-in GT strategy can be used when the expected phenotype of the resulting mutant is morphologically distinguishable from that of the transformation background. In this strategy, the GT vector does not carry a resistance marker and the complementing DNA is flanked by the 5′ and 3′ TSs. After transformation with the resistance marker-free vector, transformed mutants harboring single copy on-locus GT events can easily be identified based on the phenotype. The use of this approach offers two advantages; elimination of the time-consuming Cre/loxP-mediated transformation step to remove the resistance marker and no introduction of *loxP*-associated mutations affecting splicing integrity of the targeted intron.

Finally, in this study we investigated the functional significance of highly conserved amino acid residues in the DEK1-LG3 domain by performing site-directed mutagenesis using the In23-5′ strategy to knock-in mutated versions of *LG3* into the *dek1Δlg3* mutant. *HRC*-free *PpLG3-M1*, *PpLG3-M2* and *PpLG3-M3* mutant lines, harboring substitution of amino acids E^1477^Q^1478^(M1), E^1481^(M2) and S^1497^(M3) to alanine, respectively, all developed wild-type like gametophores indicating that the targeted amino acids are not critically important for DEK1-LG3 function during gametophore development. Also, worth noting is that we have observed that transformation of wild-type protoplasts with constructs designed to introduce site-directed amino acid substitutions may result in selection of marker-resistant plants harboring the wild-type sequence at the targeted position. Therefore, we recommend the use of deletion mutants (lacking the sequence for which the substitution is to be made), rather than wild-type plants, as the transformation background for creating mutants harboring single (or few) amino acid substitutions.

## Methods

### Plant material, growth conditions and transformation

The *Physcomitrella patens* Gransden strain and the *P*. *patens dek1Δlg3* mutant^29^ were used throughout this study. Tissue maintenance and production was performed on BCDA medium as described by Cove and co-workers^43^. *P*. *patens* tissue and protoplasts were grown under long day conditions (16 hours light [70 to 80 µmolm^−2^ s^−1^]/8 hours dark) at 25 °C. *P*. *patens* protoplast transformation was performed as described in ref. 43. Growth medium was supplemented with 20 µgl^−1^ of Hygromycin B for selection of transformed plants. Tissue for phenotypic characterizations was grown on BCD medium. Tissue for reverse transcription PCR (RT-PCR) and Southern blot analysis was grown on BCDA medium overlaid with cellophane discs under long day conditions. The tissue was harvested after 7 days, snap-frozen in liquid nitrogen, ground in a mortar and used for RNA and DNA extraction.

### Construction of gene targeting vectors

The sequences of the primers used for vector constructions are listed in Supplementary Table S1. Plasmid cloning was performed using the In-Fusion® HD Cloning kit (Clontech Laboratories) according to the manufacturer’s instructions, unless otherwise stated. The PCR-generated fragments used in the In-Fusion reactions were amplified with Thermo Scientific® Phusion High-Fidelity DNA Polymerase. To verify the constructed vectors, restriction digestion and DNA sequencing analyses were performed. All nucleotide (nt) numberings are relative to the A^1^TG start site of the *P*. *patens* (*Pp*) *DEK1* genomic sequence (Pp3c17_17550; www.phytozome.net), unless otherwise stated.

Knock-out vectors: Construction of the pBHRF∆LinkerG1 vector is described by Johansen *et al*.^29^. In short, fragments spanning nt 7249–8346 and nt 11545–1254 as the 5′ and 3′ TSs, respectively, flank the hygromycin resistance cassette in pBHRF∆LinkerG1 (Fig. 2a). GenScript Inc. (USA) performed fragment synthesis and cloning of the pBHRFΔLinkerG2 vector. The following fragments were synthesized de novo: the 5′ TS, nt 7249–8404 flanked by HindIII and NruI recognition sequences and the 3′ TS, a mutagenized fragment spanning from nt 8402–12893 with a 3257 bp deletion from nt 8462–11718 and flanked by 5′ SpeI and 3′ NsiI recognition sequences. The 5′ and 3′ TSs were cloned sequentially into pBHRF^20^ using HindIII/NruI and SpeI/NsiI restriction enzyme pairs, respectively, resulting in vector pBHRF∆LinkerG2 (Fig. 2b). Prior to *P*. *patens* protoplast transformation, the pBHRF∆LinkerG1 and pBHRF∆LinkerG2 vectors were digested with the restriction enzymes BmrI and PacI.

Knock-in vectors: The vectors to re-insert WT and heterologous DEK1-LG3 sequences into the *DEK1ΔLG3* locus in the *dekΔlg3* mutant using the In23-3′ strategy (Fig. 3) were constructed from vector pBHRF∆LinkerG1^29^. To make the vector containing the WT LG3 sequence, nt 8347–11544 was first PCR-amplified using primers SP_Inf_6 N and ASP_Inf_6 N from genomic *P*. *patens* DNA. The resulting PCR product was mixed with linearized (at nt position 8346) pBHRF*Δ*LinkerG1 DNA, produced by PCR using primers SP_Inf_5 N and ASP_Inf_5 N. The linearized vector and insert were ligated using an In-Fusion reaction, resulting in the WT LG3 complementation vector pBHRF_PpLG3_In23-3′ (Fig. 3a). To make the construct for DEK1 LG3 cross-species complementation tests, the WT genomic *DEK1 LG3* sequences from *Arabidopsis thaliana* (*At*) (nt 6741–7450; relative to the A^1^TG start site in AT1G55350) and *Marchantia polymorpha* (*Mp*) (nt 1258573–1259287; scaffold 26, JGI scaffold genomic data v3.1) were first PCR-amplified using primer sets MpLG3_Inf-F/MpLG3_Inf-R and AtLG3_Inf-F/AtLG3_Inf-R, respectively. Vector pBHRF_PpLG3_In23-3′ was PCR-amplified to delete nt 10567–11239, corresponding to the *DEK1 LG3* sequence, using primers SP_Inf_2 and ASP_Inf_2X. Finally, the linearized vector and the *AtLG3* and *MpLG3* amplicons were ligated in two separate In-Fusion reactions creating the cross-species complementation vectors pBHRF_AtLG3_In23-3′ and pBHRF_MpLG3_In23-3′, respectively. The vectors to re-insert *P*. *patens* DEK1 LG3 WT and mutated sequences using the In23-5′ strategy (Fig. 3b) were built from pBHRF^20^. First, the 3′ TS (nt 11334–12511) was PCR-amplified using primers 3TS-F and 3TS-R. The resulting product was cloned into linearized plasmid pBHRF, produced by PCR using primers pBHRF-F and pBHRF-R, resulting in plasmid pBHRF_3′ TS. The 5′ TS (*DEK1* nt 9568–11333), which harbors the *LG3* coding sequence, was PCR-amplified from genomic *P*. *patens* DNA using primers 5TS-F and 5TS-R and subsequently cloned into the pCR-Blunt vector (Invitrogen) to obtain the pCR_5′ TS vector. To make mutated versions of the *LG3* sequence, the pCR_5′ TS plasmid was subjected to mutagenesis reactions using the GeneArt® Site-Directed Mutagenesis PLUS kit (Invitrogen) with the mutagenic primer pairs M1-F/M1-R, M2-F/M2-R and M3-F/M3-R, resulting in three mutated 5′ TSs containing the M1 (E1477A;Q1478A), M2 (E^1481^A) and M3 (S^1497^A) substitutions, respectively. The WT and mutagenized versions of the 5′ TS were each amplified with primers 5TS_Inf-F and 5TS_Inf-R, and sub-cloned into XhoI-linearized vector pBHRF_3′ TS, resulting in the generation of the complementation vectors pBHRF_PpLG3_In23-5′, pBHRF_PpLG3_M1_In23-5′ pBHRF_PpLG3_M2_In23-5′ and pBHRF_PpLG3_M3_In23-5′. Prior to *P*. *patens* protoplast transformation, these vectors were digested with the restriction enzymes BmrI and PacI.

### PCR genotyping of transformants

Each mutant was subjected to three rounds of PCR genotyping using the Phire Plant Direct PCR kit (Thermo Fisher Scientific) according to the manufacturer’s instructions. The sequences of the primers used for genotyping each set of transformants are provided in Supplementary Table S1. For primary genotyping, knock-out and knock-in mutants were genotyped for the loss and gain of genomic sequence, respectively. Secondary genotyping PCR was performed to verify proper 5′ and 3′ targeting at the locus using a primer pair annealing within the DNA fragment used for transformation and 5′ or 3′ to the 5′ and 3′ TSs, respectively. Finally, tertiary genotyping PCR, mutants harboring single-copy insertion events at the locus were identified using primers annealing 5′ and 3′ to the 5′ and 3′ TSs, respectively.

For *dek1∆linkerG1* and *dek1∆linkerG2*, primary genotyping was performed using primers Linker-F and Linker-R. Lines without a detectable PCR product were subjected to the second PCR using primer pairs Linker-2F and 35S_rev1 for the 5′ targeting and, Term-fwd and Linker-2R for the 3′ targeting. Lines with both 5′ and 3′ targeting were assessed for single copy insertion at each locus using the primers Linker-2F and Linker 2 R. For the In23-3′ lines, gain of sequence was assessed using primers ARMSeq 6 and ASP_PpARM_inf 1. Positive lines were genotyped for on-locus targeting using primers TM2Seq1 and 35S_rev1 for 5′ targeting, and Term_fwd and Ex30 R for 3′ targeting. Single copy insertion was assessed using TM2seq1 and Ex30 R. For In23-5′ lines, primary genotyping was performed using primers LG3_fwd and LG3_rev; secondary genotyping: ARMSeq3 and 35S_rev1 for 5′ targeting and, Term_fwd and Ex30 R for 3′ targeting, respectively. Tertiary genotyping was performed using primers ARMSeq3 and Ex30 R.

### Molecular characterization of transformants

Genomic DNA for Southern Blot analysis was extracted using the Nucleon™ PhytoPure™ Genomic DNA Extraction Kit (GE Healthcare). Southern Blot was performed as described^19^ using ca. 1 µg DNA per digestion. Probes were DIG-labelled using the DIG Probe PCR synthesis kit (Roche, Indianapolis, USA) according to the manufacturer’s instructions. pBHRF∆LinkerG1 DNA was used as template for PCR amplification of the TS and hygromycin-resistance probes; primers for amplification of the; 5′ TS probe: Δarm5′-fwd and Δarm5′-rev; 3′ TS probe: Δarm3′-fwd and Δarm3′-rev; HRC probe: HRC-fwd and HRC-rev. The sequences of the primers used to generate the probes are provided in Supplementary Table S1.

RT-PCR and DNA sequencing were employed to amplify and analyze the *DEK1* cDNA to verify proper deletion/insertion and splicing of the transcript. Total RNA was isolated from protonema tissue using the Plant RNeasy Kit (Qiagen). Five hundred ng DNaseI-treated total RNA was reverse-transcribed using 100 U Superscript™ III Reverse Transcriptase (Invitrogen) primed with 200 ng random hexamers at 55 °C for 60 min. Phusion® High-Fidelity DNA polymerase was used to amplify the DEK1 cDNA target using primers TM2Seq1 and ASP_PpCALP, annealing in exons 11 and 27, respectively. After Exo-SAP treatment to remove unincorporated primers, the PCR product was sequenced with appropriate primers using BigDye® v3.1 chemistry (ABI) according to the SteP method^44^. Extension products were precipitated using sodium-acetate/ethanol, dissolved in Hi-Di™ Formamide (ABI) and finally sequenced by Capillary Electrophoresis using the 3130xL Genetic Analyzer. DNA sequences were analyzed using Genomic Workbench v7.

### Microscopy

The mutants were observed by light microscopy.

## Electronic supplementary material

Supplementary Information

## References

[1] Wilson RC, Doudna JA (2013). Molecular mechanisms of RNA interference. Annual review of biophysics.

[2] Lange M, Yellina AL, Orashakova S, Becker A (2013). Virus-induced gene silencing (VIGS) in plants: an overview of target species and the virus-derived vector systems. Methods in molecular biology (Clifton, N.J.).

[3] Kurowska M (2011). TILLING - a shortcut in functional genomics. Journal of Applied Genetics.

[4] Bortesi L, Fischer R (2015). The CRISPR/Cas9 system for plant genome editing and beyond. Biotechnology advances.

[5] Schaefer DG (2001). Gene targeting in Physcomitrella patens. Current opinion in plant biology.

[6] Britt AB, May GD (2003). Re-engineering plant gene targeting. Trends Plant Sci.

[7] Hanin M, Paszkowski J (2003). Plant genome modification by homologous recombination. Current opinion in plant biology.

[8] Rothstein R (1991). Targeting, disruption, replacement, and allele rescue: integrative DNA transformation in yeast. Methods in enzymology.

[9] Deng C, Capecchi MR (1992). Reexamination of gene targeting frequency as a function of the extent of homology between the targeting vector and the target locus. Mol Cell Biol.

[10] Sodeinde OA, Kindle KL (1993). Homologous recombination in the nuclear genome of Chlamydomonas reinhardtii. Proceedings of the National Academy of Sciences.

[11] Collonnier C (2016). CRISPR-Cas9-mediated efficient directed mutagenesis and RAD51-dependent and RAD51-independent gene targeting in the moss Physcomitrella patens. Plant Biotechnol J.

[12] Lopez-Obando, M. *et al*. Simple and Efficient Targeting of Multiple Genes Through CRISPR-Cas9 in Physcomitrella patens. *G3* (*Bethesda*, *Md*.), doi:10.1534/g3.116.033266 (2016). 10.1534/g3.116.033266 PMC5100863 27613750

[13] King BC (2016). *In vivo* assembly of DNA-fragments in the moss, Physcomitrella patens. Scientific reports.

[14] Gibson DG (2008). Complete chemical synthesis, assembly, and cloning of a Mycoplasma genitalium genome. Science (New York, N.Y.).

[15] Shao Z, Zhao H, Zhao H (2009). DNA assembler, an *in vivo* genetic method for rapid construction of biochemical pathways. Nucleic acids research.

[16] Nandi AK, Roginski RS, Gregg RG, Smithies O, Skoultchi AI (1988). Regulated expression of genes inserted at the human chromosomal beta-globin locus by homologous recombination. Proceedings of the National Academy of Sciences of the United States of America.

[17] Charlot F (2014). RAD51B plays an essential role during somatic and meiotic recombination in Physcomitrella. Nucleic acids research.

[18] Kamisugi Y (2012). MRE11 and RAD50, but not NBS1, are essential for gene targeting in the moss Physcomitrella patens. Nucleic acids research.

[19] Perroud PF, Quatrano RS (2006). The role of ARPC4 in tip growth and alignment of the polar axis in filaments of Physcomitrella patens. Cell motility and the cytoskeleton.

[20] Schaefer DG (2010). RAD51 loss of function abolishes gene targeting and de-represses illegitimate integration in the moss Physcomitrella patens. DNA repair.

[21] Spinner L (2010). The function of TONNEAU1 in moss reveals ancient mechanisms of division plane specification and cell elongation in land plants. Development.

[22] Wu SZ, Ritchie JA, Pan AH, Quatrano RS, Bezanilla M (2011). Myosin VIII regulates protonemal patterning and developmental timing in the moss Physcomitrella patens. Molecular plant.

[23] Trouiller B, Schaefer DG, Charlot F, Nogue F (2006). MSH2 is essential for the preservation of genome integrity and prevents homeologous recombination in the moss Physcomitrella patens. Nucleic acids research.

[24] Uenaka H, Kadota A (2007). Functional analyses of the Physcomitrella patens phytochromes in regulating chloroplast avoidance movement. The Plant Journal.

[25] Dunø M, Bendixen C, Krejci L, Thomsen B (1999). Targeted deletions created in yeast vectors by recombinational excision. Nucleic acids research.

[26] Silva JC, Borges JC, Cyr DM, Ramos CHI, Torriani IL (2011). Central domain deletions affect the SAXS solution structure and function of Yeast Hsp40 proteins Sis1 and Ydj1. BMC Structural Biology.

[27] Henderson DM, Belanto JJ, Li B, Heun-Johnson H, Ervasti JM (2011). Internal deletion compromises the stability of dystrophin. Human molecular genetics.

[28] Mbikay M (2007). A targeted deletion/insertion in the mouse Pcsk1 locus is associated with homozygous embryo preimplantation lethality, mutant allele preferential transmission and heterozygous female susceptibility to dietary fat. Developmental Biology.

[29] Johansen W (2016). The DEK1 calpain Linker functions in three-dimensional body patterning in Physcomitrella patens. Plant physiology.

[30] Perroud PF (2014). Defective Kernel 1 (DEK1) is required for three-dimensional growth in Physcomitrella patens. The New phytologist.

[31] Wang C (2003). The calpain domain of the maize DEK1 protein contains the conserved catalytic triad and functions as a cysteine proteinase. The Journal of biological chemistry.

[32] Schuler MA (2008). Splice site requirements and switches in plants. Current topics in microbiology and immunology.

[33] Szcześniak MW, Kabza M, Pokrzywa R, Gudyś A, Makałowska I (2013). ERISdb: A Database of Plant Splice Sites and Splicing Signals. Plant and Cell Physiology.

[34] Simpson CG (2002). Mutational analysis of a plant branchpoint and polypyrimidine tract required for constitutive splicing of a mini-exon. RNA (New York, N.Y.).

[35] Goodall GJ, Filipowicz W (1989). The AU-rich sequences present in the introns of plant nuclear pre-mRNAs are required for splicing. Cell.

[36] Yusa, K. *et al*. Targeted gene correction of alpha(1)-antitrypsin deficiency in induced pluripotent stem cells. *Nature* **478**, 391-+, doi:10.1038/nature10424 (2011). 10.1038/nature10424 PMC3198846 21993621

[37] Nishizawa-Yokoi A, Endo M, Ohtsuki N, Saika H, Toki S (2015). Precision genome editing in plants via gene targeting and piggyBac-mediated marker excision. The Plant journal: for cell and molecular biology.

[38] Nishizawa-Yokoi A, Endo M, Osakabe K, Saika H, Toki S (2014). Precise marker excision system using an animal-derived piggyBac transposon in plants. The Plant journal: for cell and molecular biology.

[39] Cary LC (1989). Transposon mutagenesis of baculoviruses - analysis of trichoplusia-ni transposon ifp2 insertions within the fp-locus of nuclear polyhedrosis viruses. Virology.

[40] Ding S (2005). Efficient transposition of the piggyBac resource (PB) transposon in mammalian cells and mice. Cell.

[41] Rensing SA, Fritzowsky D, Lang D, Reski R (2005). Protein encoding genes in an ancient plant: analysis of codon usage, retained genes and splice sites in a moss, Physcomitrella patens. BMC Genomics.

[42] Perroud PF, Quatrano RS (2008). BRICK1 is required for apical cell growth in filaments of the moss Physcomitrella patens but not for gametophore morphology. Plant Cell.

[43] Cove DJ (2009). The moss Physcomitrella patens: a novel model system for plant development and genomic studies. Cold Spring Harbor protocols.

[44] Platt AR, Woodhall RW, George AL (2007). Improved DNA sequencing quality and efficiency using an optimized fast cycle sequencing protocol. BioTechniques.

