# Supplementary material for: An intragenic mutagenesis strategy in Physcomitrella patens to preserve intron splicing
Source: Sci Rep. 2017 Jul 11;7:5111. doi: 10.1038/s41598-017-05309-w (PMC5505980; doi:10.1038/s41598-017-05309-w)
Supplement: Supplementary file 1 — Supplementary Information [file 41598_2017_5309_MOESM1_ESM.pdf]

An intragenic mutagenesis strategy in *Physcomitrella patens* to preserve intron splicing.

Ako Eugene Ako, Pierre-François Perroud, Joseph Innocent, Viktor Demko, Odd-Arne Olsen and Wenche Johansen

SUPPLEMENTARY INFORMATION

Supplementary Figure S1. *dek1Δlinker* knock-out mutant phenotypes.

Supplementary Figure S2. Molecular characterization of the *dek1Δlinker* knock-out mutants.

Supplementary Figure S3. Vector construction, targeted insertion of *DEK1-LG3*, molecular and phenotypic characterization of mutant plants.

Supplementary Table S1. List of oligonucleotide primers and their sequences used in this study.

Supplementary Figure S1

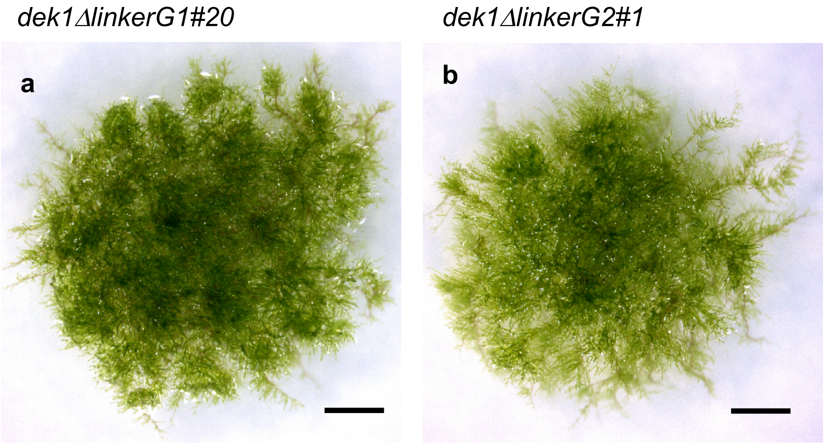

Figure S1. ***dek1Δlinker* knock-out mutant phenotypes**. Phenotype of 3-week-old *dek1ΔlinkerG1#20* and *dek1ΔlinkerG2#1* mutant plants harboring the *HRC*; the plants display filamentous growth without gametophores. Scale bar: 2 mm.

Supplementary Figure S2

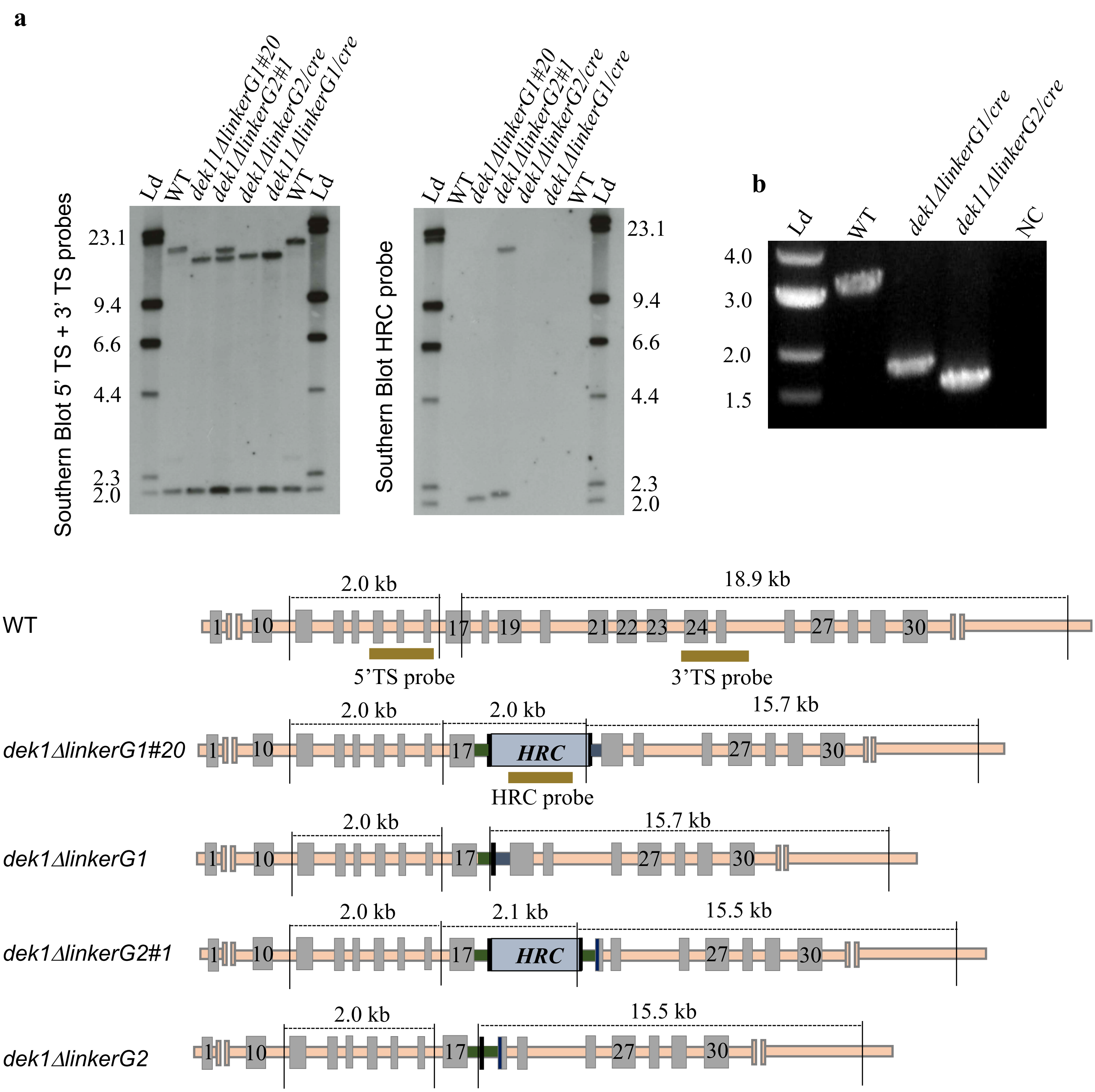

Figure S2. **Molecular characterization of *dek1Δlinker* knock-out mutants**. (**a**) Southern blot analysis using a mixture of 5' and 3' TS probes (left blot) and the HRC probe (right blot) shows the expected band sizes (as depicted in the schematics below) with no sign of off-locus integration. The analysis identifies the *dek1ΔlinkerG1#20* as a single copy line and *dek1ΔlinkerG2#1* as a multicopy line. After Cre/lox-mediated *HRC* removal, both strains show loss of the *HRC* and no sign of re-integration (right blot). The schematics show the exon-intron structure of the mutants analyzed in the Southern blot. The grey and blue boxes correspond to the exons of the *P. patens* gene; introns are colored in orange. The 5' and 3' targeting sequences (TS) are boxed. The *loxP* sites are shown as vertical black lines (bars). The hybridization sites of the 5' TS, 3' TS and *HRC* probes are indicated by brown bars in the schematic. The black vertical lines represent the restriction sites of the enzyme *Bgl*II used for DNA digesting (**b**) RT-PCR analysis of the *dek1ΔlinkerG1/cre* and *dek1ΔlinkerG2/cre* showing a single PCR amplification product of the expected sizes of 1.7 kb for *dek1ΔlinkerG1/cre* and 1.5 kb for *dek1ΔlinkerG2/cre*.

Supplementary Figure S3

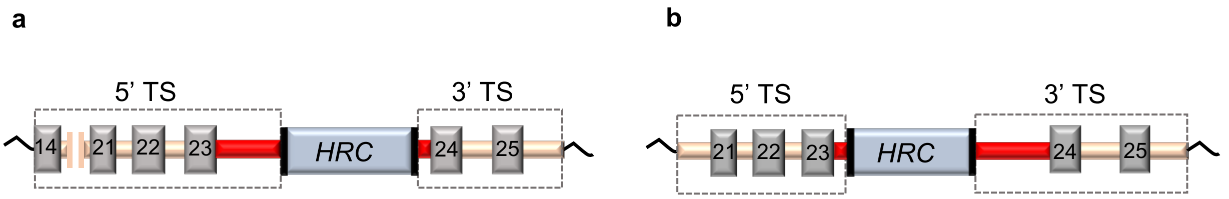

Figure S3. ***DEK1 LG3* knock-in.** Schematic representation of vector **(a)** *In23-3'* and **(b)** *In23-5'*. The grey boxes correspond to exons in the *P. patens* gene. Introns are colored in orange, with the exception of intron 23 (red), which is enlarged to illustrate the difference in the position of the *HRC* within the intron. The 5' and 3' targeting sequences (TS) are boxed. The *loxP* sites are shown as vertical black lines. The numbers correspond to the exons of the *DEK1* ORF. **(c)** cDNA sequencing chromatogram of the (i) *PpLG3-3'/cre*, (ii) *AtLG3/cre* and (iii) *MpLG3/cre* mutant *DEK1* transcripts, showing insertion of the wild type *PpDEK1-LG3* (i) and heterologous *AtDEK1-LG3* (ii) and *MpDEK1-LG3* (iii) sequences into the *DEK-LG3* locus of the *dek1Δlg3* mutant. Mis-splicing of the *AtLG3/cre DEK1* pre-mRNA (ii) results in a loss of 64 nucleotides (nt.) corresponding to the last 61 nt of the *AtDEK1* exon 21 (nt. 4514 - 4574) and the first 3 nt. of the *AtDEK1* exon 22 (nt. 4575 - 4577), leading to the introduction of a premature stop codon (red box). The native *MpLG3* sequence (iii) is correctly spliced. **(d)** Phenotype of 3-week-old *AtLG3/cre* and *MpLG3/cre* plants showing filamentous growth without gametophores. **(e)** cDNA sequencing chromatogram of M1: *PpLG3-M1/cre*, M2: *PpLG3-M2/cre* and M3: *PpLG3-M3/cre* mutant *DEK1* transcripts showing the presence of the introduced mutations (red box with nucleotides in lower case). **(f)** Phenotype of 4-week-old colonies (upper panel) and developed gametophores (lower panel) of *PpLG3-M1/cre*, *PpLG3-M2/cre*, *PpLG3-M3/cre* and *dek1Δlg3* mutant plants. In contrast to the *dek1Δlg3* mutant plant, which displays narrow phyllids, *PpLG3-M1/cre*, *PpLG3-M2/cre* and *PpLG3-M3/cre* mutant plants develop gametophores with fully developed phyllids. Scale bar: upper pictures = 2 mm, lower pictures = 500 μm.

Supplementary Figure S3c(i) – Insertion of *PpDEK1-LG3* into the *DEKΔLG3* locus.

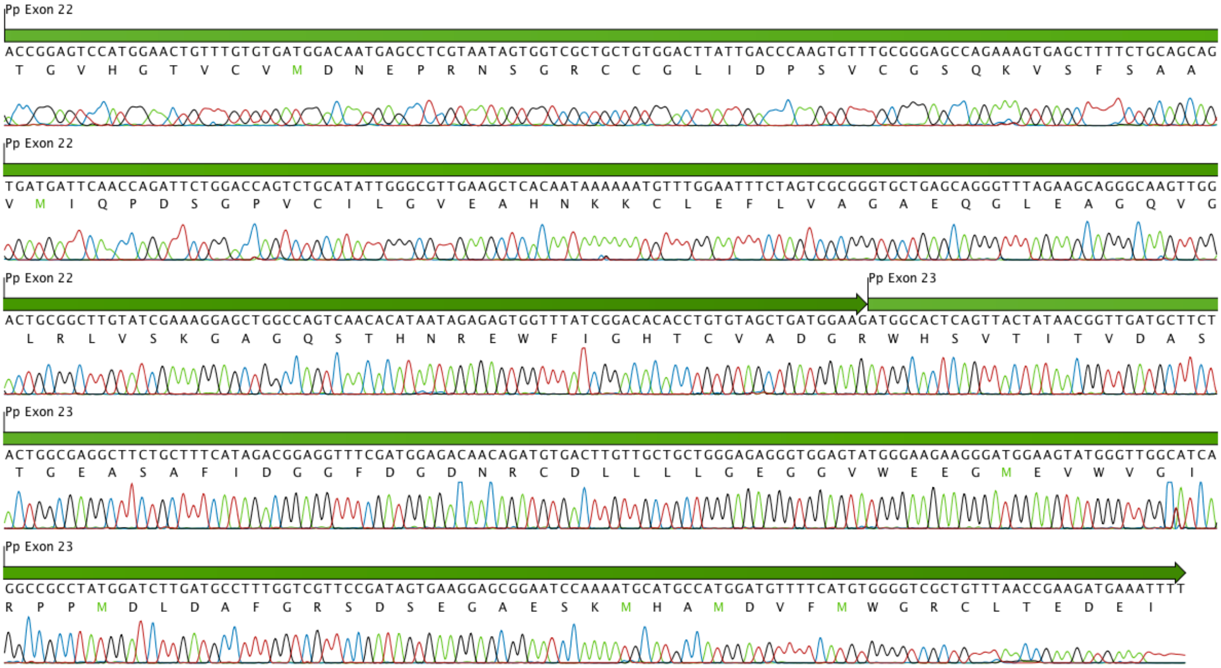

Supplementary Figure S3c(ii) – Insertion of *AtDEK1-LG3* into the *DEKΔLG3* locus.

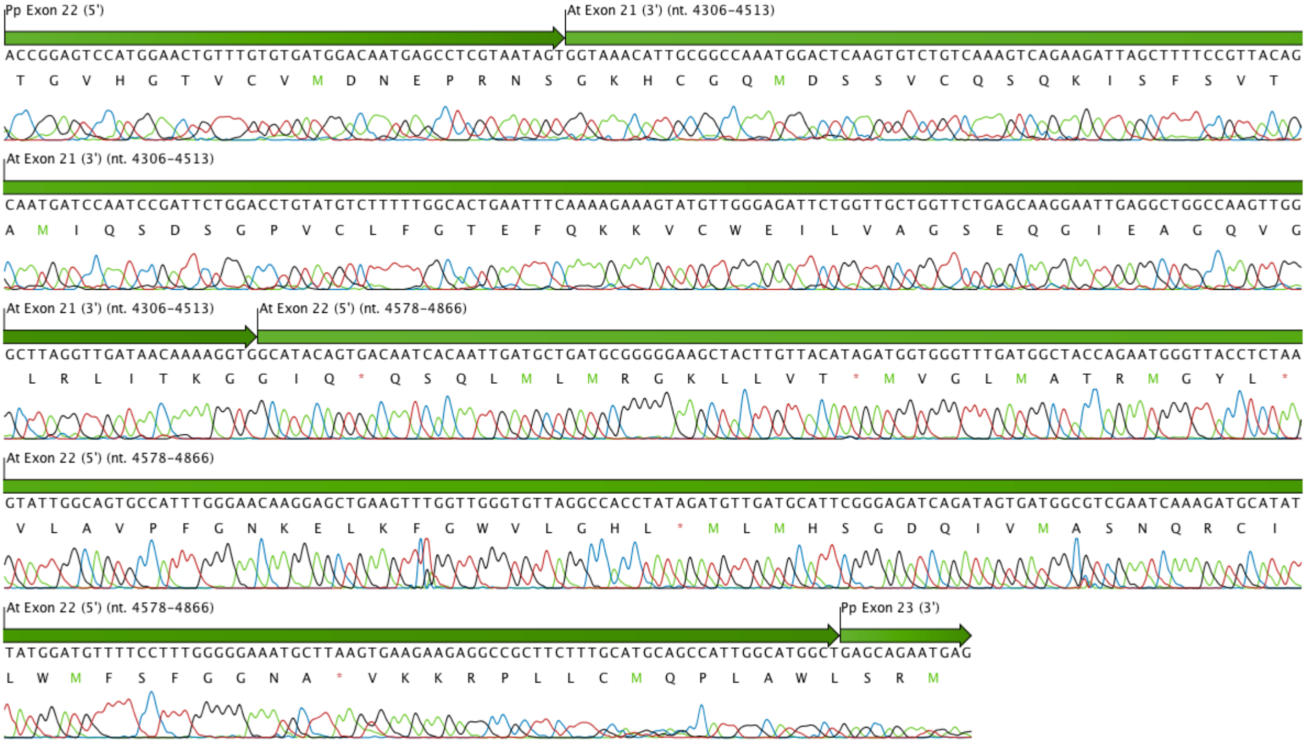

Supplementary Figure S3c(iii) – Insertion of *MpDEK1-LG3* into the *DEKΔLG3* locus.

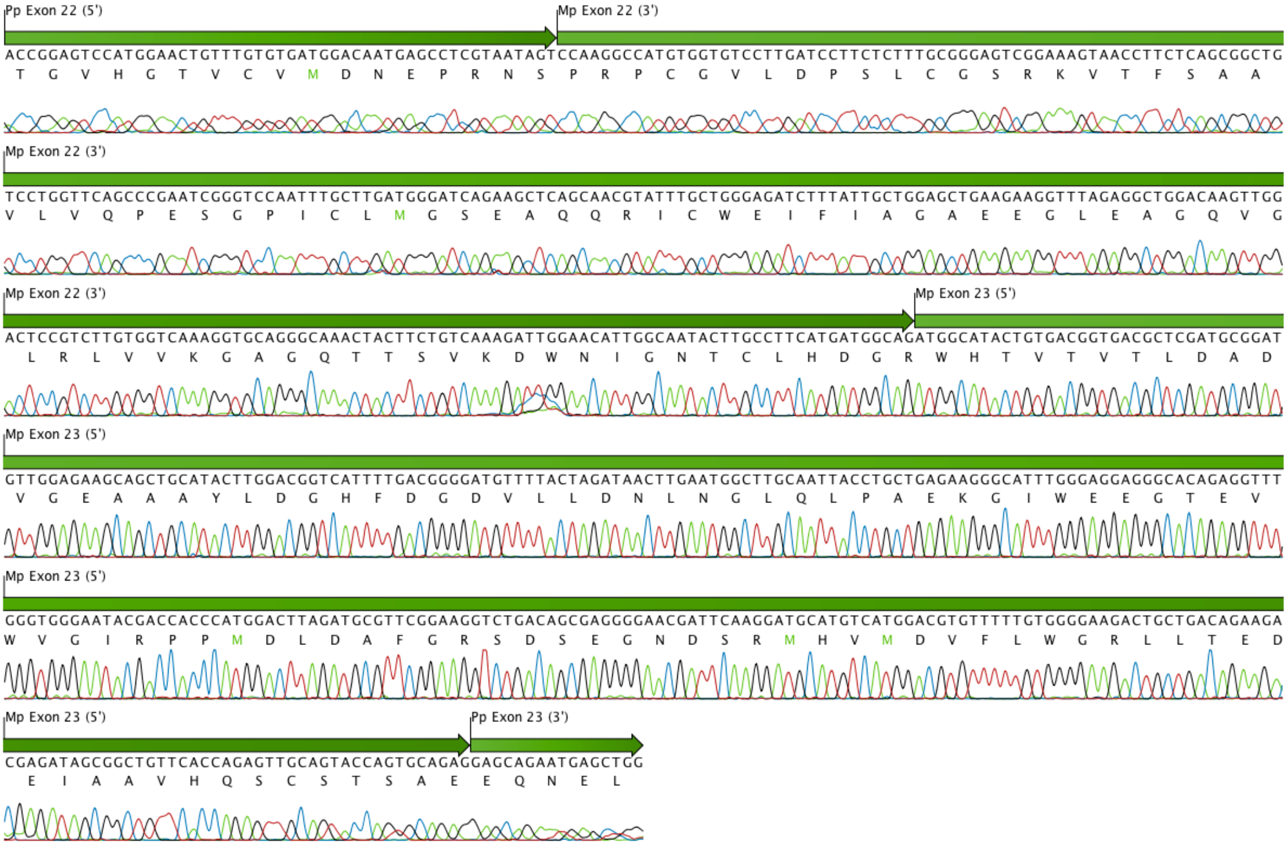

Supplementary Figure S3

d

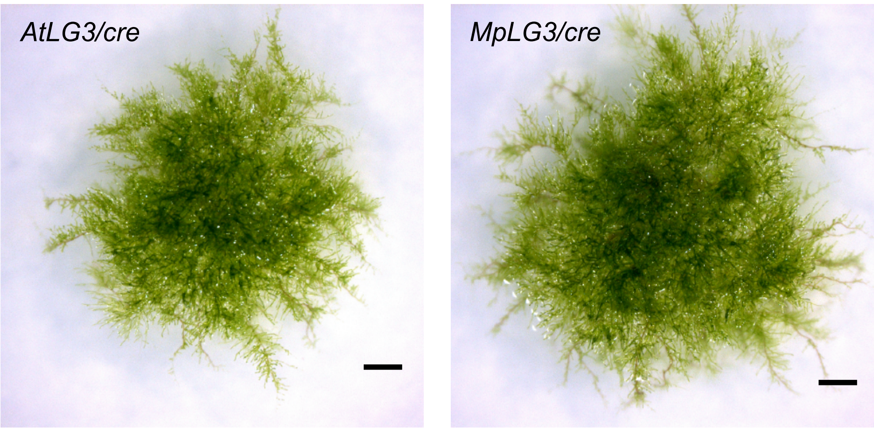

e

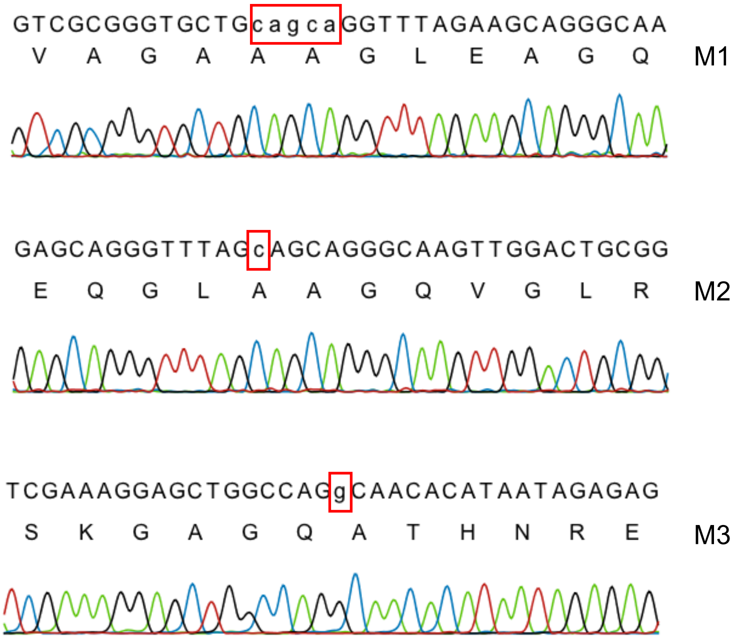

f

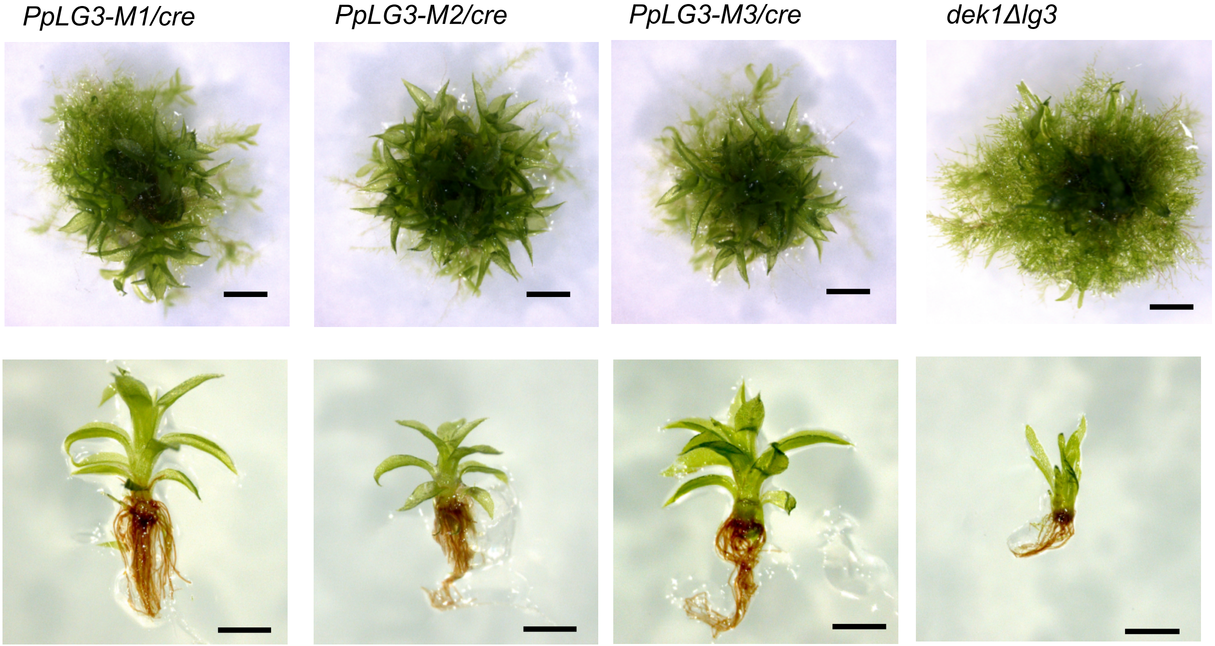

Supplementary Table S1: List of oligonucleotide primers and their sequences used in this study.

**Primer name**	**Oligonucleotide sequence (5’ → 3’)**
ARMSeq 6	TGCAGGTACCAAAGAAGCAGC
ASP_PpArm_inf1	ATAGATACACGACCGGCAG
TM2Seq1	TACCTAGGGTGGGCAATTGC
35S_rev1	GGAGCCACCTTCCTTTTCCA
Term_fwd	AGGGTTCTTATAGGGTTTCGCTCATG
LG3_fwd	TTGGCACATTTCAGACCGGA
LG3_rev	TTCCATTGGAGATCCCTGCC
ARMSeq3	TGTTTTAGCACGGCTATTCTTTTC
ASP_PpCALP	TCAATCTCCTCTCCAGCACCT
5TS-F	TACGTCGCGACTCGATGCATGATTAATATGATCTTCA
5TS-R	ACGAAGTTATCTCGACATGTGCTTCGTGATATGC
3TS-F	CGCCACGCGTGATATGTTTAATTGAGTCAGTAATTAG
3TS-R	ATGTTAACATGCATGACTGATACGTAAACGAAGATA
pBHRF-F	CATGCATGTTAACATCGATCCATGG
pBHRF-R	ATATCACGCGTGGCGCCACTAG
M1-F	TAGTCGCGGGTGCTGCAGCAGGTTTAGAAGCAGG
M1-R	CCCTGCTTCTAAACCTGCTGCAGCACCCGCGACTA
M2-F	CTGAGCAGGGTTTAGCAGCAGGGCAAGTTGG
M2-R	CCAACTTGCCCTGCTGCTAAACCCTGCTCAG
M3-F	AAAGGAGCTGGCCAGGCAACACATAATAGAG
M3-R	CTCTATTATGTGTTGCCTGGCCAGCTCCTTT
5TS_Inf-F	TACGTCGCGACTCGATGCATGATTAATATGATCTTCA
5TS_Inf-R	ACGAAGTTATCTCGACATGTGCTTCGTGATATGC
AtLG3_inf-F	GAGCCTCGTAATAGTGGTAAACATTGCGGCCAAA
AtLG3_inf -R	CAGCTCATTCTGCTCAGCCATGCCAATGGCTGC
MpLG3_inf-F	GAGCCTCGTAATAGTCCAAGGCCATGTGGTGTC
MpLG3_inf-R	CAGCTCATTCTGCTCCTCTGCACTGGTACTGCA
Linker-F	TGGTTTACCTCACATTCATAT
Linker-R	GCAGGACATCAAAACACAC
SP_inf_2	GAGCAGAATGAGCTGGATATTATGG
ASP_inf _2X	ACTATTACGAGGCTCATTGTCCATC
Linker-2F	TCTGTGGAGGATCTTACGTGGGC
Linker-2R	CCCACTCACCCTGCATGCATTA
Ex30R	TGTGGTTGGAACAATCGTGTAAC
SP_Inf_6N	TGACATTATTACTATTGGTTTACCTCACATTCATAT
ASP_Inf_6N	TTATCTCGAGTCGCGGCAGGACATCAAAACACAC
SP_Inf_5N	CGCGACTCGAGATAACTTCG
ASP_Inf_5N	ATAGTAATAATGTCATATGCGTACACCT
Δarm_5’ fwd	TCTAGCTTTTGTGATGACAGGTTGGC
Δarm_ 5’ rev	AAACTCGCAACCACTCCCCATC
Δarm_3’ fwd	GTTTGAGGATTGGAGATATGAT
Δarm_3’ rev	AATGGACTACAAACTGATACG
HRC-F	CTCCAGAAGAAGATGTTGG
HRC-R	ATGAAAAAGCCTGAACTCAC
